# Extensive Inter-Domain Lateral Gene Transfer in the Evolution of the Human Commensal *Methanosphaera stadtmanae*

**DOI:** 10.3389/fgene.2012.00182

**Published:** 2012-09-19

**Authors:** Mor Nadia Lurie-Weinberger, Michael Peeri, Tamir Tuller, Uri Gophna

**Affiliations:** ^1^Department of Molecular Microbiology and Biotechnology, George S. Wise Faculty of Life Sciences, Tel Aviv UniversityTel Aviv, Israel; ^2^Department of Cell Research and Immunology, George S. Wise Faculty of Life Sciences, Tel Aviv UniversityTel Aviv, Israel; ^3^Department of Biomedical Engineering, Faculty of Engineering, Tel Aviv UniversityTel Aviv, Israel

**Keywords:** horizontal gene transfer, microbial evolution, archaeal genomics, archaea, methanogens, human gut

## Abstract

*Methanosphaera stadtmanae* is a commensal methanogenic archaeon found in the human gut. As most of its niche-neighbors are bacteria, it is expected that lateral gene transfer (LGT) from bacteria might have contributed to the evolutionary history of this organism. We performed a phylogenomic survey of putative LGT events in *M. stadtmanae*, using a phylogenetic pipeline. Our analysis indicates that a substantial fraction of the proteins of *M. stadtmanae* are inferred to have been involved in inter-domain LGT. Laterally acquired genes have had a large contribution to surface functions, by providing novel glycosyltransferase functions. In addition, several ABC transporters seem to be of bacterial origin, including the molybdate transporter. Thus, bacterial genes contributed to the adaptation of *M. stadtmanae* to a host-dependent lifestyle by allowing a larger variation in surface structures and increasing transport efficiency in the gut niche which is diverse and competitive.

## Introduction

Lateral gene transfer (LGT) is an important force in microbial evolution and a major source of genetic variation and innovation (Doolittle et al., 2003). Although LGT between Archaea and Bacteria is considered relatively rare, genomes of microorganisms that share a niche almost exclusively with members of the other domain were shown to have acquired many genes by inter-domain transfer. For example, nearly a third of the genes of the methanogenic archaeon *Methanosarcina mazei* have their closest homologs in bacteria (Deppenmeier et al., 2002), and a large fraction of the genome of the bacterium *Thermotoga maritima* has an archaeal origin (Nelson et al., 1999).

*Methanosphaera stadtmanae* is a methanogenic archaeon that is a human commensal (Miller and Wolin, 1985) found mostly in the colon, but has also been found in dental plaque (Belay et al., 1988). *M. stadtmanae* and its closest phylogenetic neighbor, *Methanobrevibacter smithii*, represent the only two archaeal species commonly found in humans (Fricke et al., 2006). *M. stadtmanae* has the most restricted energy metabolism of all methanogenic archaea: it can only generate methane by reduction of methanol with hydrogen, and is dependent on acetate as a carbon source (Fricke et al., 2006).

*M. stadtmanae* grows in a niche that is predominantly colonized by a multitude of bacterial species, especially bacteria relating to two major phyla, Firmicutes and Bacteroidetes (Eckburg et al., 2005; Samuel et al., 2007), therefore it appears likely that its genome will show traces of inter-domain LGT from bacterial species, as has been recently shown for its closest phylogenetic neighbor*, M. smithii* (Lurie-Weinberger et al., 2011). Indeed, sequencing of the *M. stadtmanae* genome has identified 323 ORFs without homologs in the genomes of other Archaea, out of which 73 had high levels of similarity to protein coding genes of either bacteria or eukaryotes (Fricke et al., 2006). Here we expand and update that assessment by whole-genome phylogenetic analysis, in order to assess the extent of inter-domain LGT in the *M. stadtmanae* genome and demonstrate its importance to the evolution of this human commensal. We accomplished this by utilizing an automatic phylogenetic pipeline, followed by a manual inspection of the resulting phylogenies. We also compare gene-family content between *M. stadtmanae* and its sister group, the *Methanobrevibacter* genus and infer which gene families were acquired by the ancestral mammalian-associated methanogen.

## Materials and Methods

### Phylogenetic analysis

We utilized the automatic pipeline PhyloGenie (Frickey and Lupas, 2004) in order to create protein-based phylogenetic trees for each protein in the *M. stadtmanae* genome. These analyses were performed against the non-redundant (nr) protein database, downloaded from NCBI with an *e*-value of 0.00001. About 1336 trees were constructed, and each tree was then manually examined in order to detect possible events of LGT. LGT candidates were defined as trees in which the closest phylogenetic neighbor of *M. stadtmanae* was not an archaeal species but a bacterial or eukaryotic one. Since the methanogenic archaeon *Methanobrevibacter* is the only other methanogen known to inhabit the human gut, trees in which *M. smithii* or *Methanobrevibacter ruminantium* were present as sister taxa were also examined, and where the two genera (*Methanobrevibacter* and *Methanosphaera*) were nested within a bacterial clade, the respective trees were also inferred to be candidates for inter-domain LGT.

A bacterial clade was defined as a clade of bacterial taxa representing at least three different phyla, and thus a sufficiently diverse sample of bacterial organisms to represent a true bacterial origin. The branch inspected (and its bootstrap support value) was therefore that of *Methanosphaera* and a bacterial homolog or of *Methanosphaera* and *Methanobrevibacter* and their closest bacterial homolog, if both were present as sister taxa on the protein tree.

### Tree reconstruction using RaxML

To verify that the inferred LGT events found with the Phylogenie pipeline [based on neighbor-joining (NJ)] were reliable, the alignments of all inferred LGT candidates were also analyzed by the maximum-likelihood method RaxML (with LG + F + G4 model). Congruence between both methods was assessed by hand and only genes for which both NJ and ML recovered a LGT tree were further considered.

### Calculations of CAI and ENC

Both codon adaptation index (CAI; Sharp and Li, 1987) and effective number of codons (ENC; Wright, 1990) calculations were performed for *M. stadtmanae* utilizing Inca 2.0 (Supek and Vlahovicek, 2004). The highly expressed ribosomal protein-encoding genes were excluded, as these are used by INCA to calculate CAI.

### Phylogenetic reconstruction of the gene-family content of the ancestor of *M. stadtmanae* and *Methanobrevibacter*

The COG data of 29 methanogenic archaea were downloaded form the NCBI FTP server, as were the COG data regarding a non-methanogenic, halophilic archaeon *Haloferax volcanii* DS2. A phylogenetic tree was constructed using the 16S ribosomal RNA sequences of these 30 species to determine their phylogenetic relationships, with *H. volcanii*_DS2 as the outgroup, using the NJ algorithm with 1000 bootstrap pseudoreplicates (see Figures 5A,B). The full list of species used can be found in Table S4 in Supplementary Material. The ancestral gene content reconstruction was based on the ancestral coevolver (ACE) algorithm (Tuller et al., 2010). The input to this algorithm was the evolutionary tree described above including its branch lengths, and information about the co-evolutionary relations between pairs of gene families. The co-evolutionary information is based on various sources of information including proximity in the protein interaction network of *E. coli* and *S. cerevisiae*, proximity in the metabolic networks of the analyzed organisms, and additional physical and functional interactions downloaded from String (http://string.embl.de/). For details see (Tuller et al., 2010). The ACE algorithm infers the ancestral COG content considering both the phylogenetic information and the co-evolutionary information.

## Results and Discussion

### Identification of lateral gene transfer candidates

To test the hypothesis that *M. stadtmanae* has had major contributions to its gene repertoire through inter-domain LGT from bacterial species, an automatic pipeline was used to generate phylogenetic trees for the entire proteome of that archaeon. Overall, 1336 trees were constructed for *M. stadtmanae* proteins (out of the 1534 annotated proteins in this organism) using the NJ method (Saitou and Nei, 1987). The trees were then manually inspected to identify putative LGT events (see Materials and Methods). The closest relative of *Methanosphaera* is *Methanobrevibacter* and these two methanogens were recovered as sister species in most of our trees. However, 137 trees had bacteria as the closest phylogenetic neighbor of *Methanosphaera*, representing possible inter-domain LGTs, while in 72 additional trees *Methanobrevibacter* was sister to *Methanosphaera*, but with the two methanogens nested within bacterial clades (in 23 cases sister taxon was *M. smithii*, in 17 *M. ruminantium*, and 32 trees had both as sister taxa). This grouping suggests that these 72 genes are the result of older lateral transfers having preceded the divergence of the two methanogenic genera. Thus 209 trees represented potential gene exchange with bacteria. An additional 14 trees had a eukaryote as the closest phylogenetic neighbor. Therefore, the total number of possible inter-domain LGT candidates found was 223, representing 16.69% of all *M. stadtmanae* trees generated in this study, and 14.54% of all proteins in its proteome (see Table S1 in Supplementary Material). All trees can be viewed at http://Methanosphaera.pazcorp.com/.

To test the validity of these results, the 223 LGT candidates genes were re-analyzed by utilizing the maximum-likelihood program RaxML. In general there was an excellent agreement between the two methods: 196 of 223 LGT (87.89%) candidate trees were also verified as such by RaxML. The remaining 22 protein phylogenies displayed a clear non-LGT phylogeny in RaxML and five alignments had too few taxa to be analyzed with RaxML (which requires at least four species). Thus, if we consider only LGT events identified by both methods, the maximum number of LGT drops to 196.

To more accurately determine the contribution of inter-domain gene transfer to the protein repertoire of *M. smithii* the direction of the transfer was inferred for each of the 223 possible LGT event indicated by NJ and the 196 detected by RaxML. Cases in which both methods indicated a possible LGT, and at least one method could be used to infer direction were considered further as lateral acquisition events.

**Figure 1:**
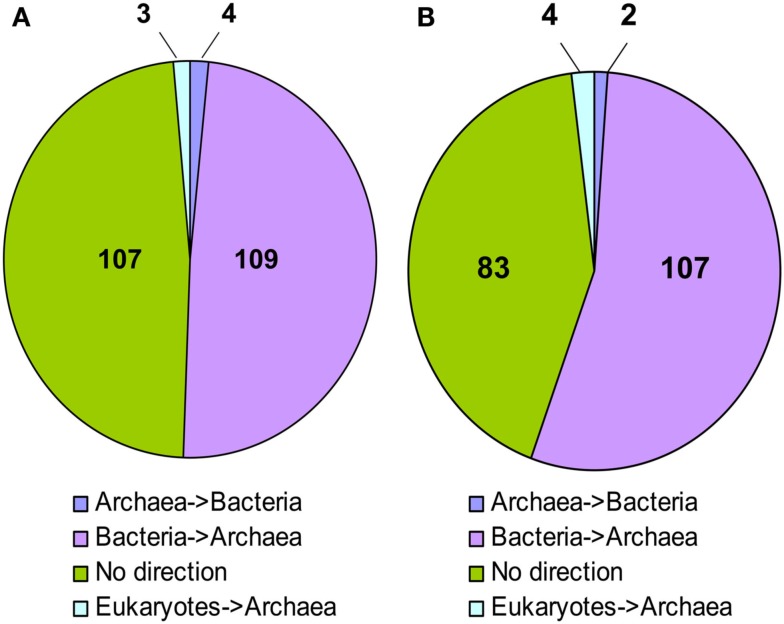
**Inferred direction of transfer of LGT candidates in *M. stadtmanae* according to (A) Neighbor-joining analysis and (B) RaxML**.

In 109 NJ trees *M. stadtmanae* was clearly nested in a bacterial clade, and thus the direction of the transfer could be inferred to be from Bacteria to Archaea. Four trees displayed the opposite direction, where LGT had occurred from Archaea into Bacteria (Msp_0101, a glycosyltransferase, Msp_0450, a serine acetyltransferase, Msp_0664, nitrogen regulatory protein P-II, and Msp_0696, a universal stress protein). Three additional trees showed possible transfer events from eukaryotes into Archaea (Msp_0130 annotated as ThsA (thermosome subunit alpha), and the two hypothetical proteins Msp_0113 and Msp_0875). No direction could be inferred for the remaining 107 proteins, representing about 48% of the trees (see Figure 1; Table S1 in Supplementary Material). It is interesting to note that of the trees in which no direction could be inferred, in 12 cases *Methanosphaera* and *Methanobrevibacter* were the only archaea present in the phylogeny, implying a likely bacterial origin of these genes, therefore the number of genes that warrant further inspection should be 121, all of which cases which represent possible transfers of bacterial origin (see Table S1 in Supplementary Material, marked “Direction unknown*”). Parallel analysis of the RaxML trees revealed that 107 of them displayed a bacterial origin, while only two showed the opposite direction, where a gene of archaeal origin was integrated into a bacterial genome, and four displayed an eukaryotic origin. The remaining 83 trees had no clear direction of transfer. Yet, in nine cases where the direction could not be inferred, *M. stadtmanae*, or *M. stadtmanae* and *M. smithii*, were the only archaea present on the tree, and thus these nine trees can also be considered LGT events (of the three additional trees mentioned above in the NJ analysis, one is now clearly of bacterial origin, another appears to show the opposite direction, and the remaining one has too few taxa for RaxML-based reconstruction).

We therefore decided to consider a given gene to be of a bacterial origin only when it was found to be an LGT candidate by both NJ and RaxML, with at least one of the methods providing an inferred direction of transfer from bacteria to archaea. This brings the total number of LGT-derived proteins of bacterial origin to 129 (107 RaxML identified candidates +13 trees whose direction could be inferred only using the original NJ method and nine of the trees where *M. smithii* and *M. stadtmanae* were the only archaea on the tree). There are also four cases of inferred LGT from Eukarya and two cases of a transfer from Archaea to Bacteria, with a total of 135 inter-domain transfer events (see Table S1 in Supplementary Material, marked in gray). LGT candidates not confirmed with RaxML (sometimes merely due to too few taxa) are not included in these numbers, as they were not considered reliable enough for further analyses.

Taking bootstrap into account, out of 109 possible LGT candidates where direction could be inferred as being from Bacteria into Archaea according to the NJ method, 17 trees had high bootstrap support (over 70), 27 trees had moderate support (51–70), and 65 had low bootstrap support (below 50). Of the 65 trees with low bootstrap support, seven trees represent cases in which *Methanosphaera* and *Methanobrevibacter* were the only archaea present in the phylogeny, implying a likely bacterial origin of these genes (see Table S1 in Supplementary Material). So, in summary, trees that can be considered to represent true LGT events are 17 trees of good bootstrap support, along with the additional 27 trees with moderate support, yet with a topology that represents a clear bacterial origin. To these trees, 11 trees where no direction could be inferred and seven trees with a bacterial origin but low bootstrap support can be added, as they display cases in which *Methanosphaera* and *Methanobrevibacter* were the only archaea present in the phylogeny (marked with asterisks in Table S1 in Supplementary Material). Therefore, there are 62 high confidence LGT candidates, and 58 candidates with lower support, totaling 120 possible LGT events inferred by NJ (see Table S1 in Supplementary Material). The same analysis performed using RaxML generated a total of 121 trees whose direction could be inferred, having different levels of bootstrap support. Of these 121 trees (Table S1 in Supplementary Material). Thirty-four were cases of high bootstrap support, 23 medium, and the remaining low. Therefore for RaxML, only 57 trees represent good bootstrap support, out of the 121 total RaxML trees where direction could be inferred, and 64 had lower support.

**Figure 2:**
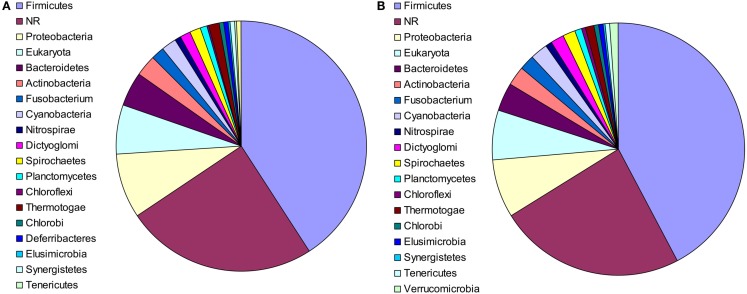
**The phylogenetic composition of the closest neighbors of LGT candidates in *M. statmanae*, based on the reconstructed trees according to (A) Neighbor-joining analysis and (B) RaxML**.

The putative bacterial lineages from which the laterally acquired genes originated were also examined. In 91 cases, the closest phylogenetic neighbor of *M. stadtmanae* was a member of the phylum Firmicutes, and in 10 cases, the closest neighbor was a member of the phylum Bacteroidetes. These results were nearly identical for RAxML, with a strong representation of Firmicutes and Bacteroidetes (82 and 7 cases, respectively). This is to be expected, as both phyla are predominant members of the intestinal microbiota of mammals (Eckburg et al., 2005; see Figure 2) and have had a long co-evolutionary history with their hosts (Ley et al., 2008). In 19 additional cases the closest phylogenetic neighbor was a member of the Proteobacteria. In 55 cases, the closest phylum could not be determined, as the relevant clade contained members of several phyla.

### LGT candidates seem to be the product of ancient transfers

The CAI and the ENC are two measures that reflect the adaptation of a coding gene to its genomic context. CAI, ranging from 0 to 1, is a measure of codon bias (Sharp and Li, 1987) and is based on the similarity between the distribution of codons in a gene and their distribution in highly expressed genes. This measure is correlated with protein expression levels (i.e., highly expressed proteins have high CAI values). On the other hand, the ENC, which ranges between 20 and 61, shows the variety of codons in a given coding gene. Genes acquired through LGT often maintain certain codons from their original host, and their codons only gradually change to better host-adapted ones by accumulation of synonymous mutations, resulting in a less biased choice of codons compared to native genes, and therefore a higher ENC. Because of their relatively less effective, foreign, codon usage, newly transferred proteins will usually display lower CAI values. Thus, a newly acquired gene will typically have a low CAI and/or a high ENC., In contrast, ancient transfers that have been ameliorated (Ochman et al., 2000) and resemble their new genomic context are expected to have ENC and CAI values that are similar to the values typical to this genome.

Indeed, most LGT candidates in *M. stadtmanae* did not differ substantially in their ENC values from their corresponding genomic averages (34.43 compared to 34.56, respectively). In general, ENC values were very diverse throughout laterally transferred genes, ranging between 26.9 and 48.87. Genes with low ENC (Values under 28.49, lower by more than 2 S.D) included the gene ThiM1, a nitroreductase, a nitrate/sulfonate/bicarbonate ABC transporter permease, a polar amino acid ABC transporter periplasmic substrate-binding protein, a transcriptional regulator, and two hypothetical proteins. On the other end of the spectrum, genes with high ENC values (values over 40.0, higher by more than two standard deviations) include a d-tyrosyl-tRNA (Tyr) deacylase, a putative secreted RNase (barnase), and three hypothetical proteins (see Table S2 in Supplementary Material).

Generally, CAI values showed the same trend as the ENC data (mean CAI of 0.72 for genes acquired from bacteria vs. a general CAI mean of 0.73), both implying that transferred genes are now highly ameliorated (see Table S2 in Supplementary Material). CAI values varied greatly among laterally transferred genes, ranging between 0.55 and 0.87. Genes with low CAI values (Values under 0.61, lower by more than 2 S.D) included an ATPase, a polar amino acid ABC transporter permease, an exopolysaccharide synthesis protein, a phosphohydrolase, and three hypothetical proteins (see Table S2 in Supplementary Material). Genes with high CAI values (values over 0.84, higher by more than 2 S.D) include a desulfoferrodoxin and ferredoxin, asn/thr-rich large protein family protein, glutamate dehydrogenase, a short chain dehydrogenase, the gene LeuD1 (a member of the Leucine biosynthetic process), ThiM1 (a member of the Thiamine biosynthesis process), and three hypothetical proteins (see Table S2 in Supplementary Material). Such high CAI values indicate high expression levels of these proteins, testifying to their functional importance.

Taken together, the ENC and CAI results imply that the vast majority of genes acquired by *M. stadtmanae* or its mammalian-associated ancestors are the products of ancient transfer events rather than recent ones. Indeed there are no plasmids or viruses in extant methanogenic archaea that can also replicate in bacteria. A relative lack of exposure of archaea to foreign bacterial DNA has been recently demonstrated in an analysis of spacers acquired by archaeal CRISPR systems (Brodt et al., 2012). Thus, one may speculate that the genetic barriers that currently restrict gene flow between bacteria in archaea had been leakier in the far past.

### Laterally acquired glycosyltransferases

Our study indicates that out of 34 annotated glycosyltransferases in the genome of *M. stadtmanae*, 18 are the result of LGT, with eight having a tree topology indicating a bacterial origin according to NJ trees, seven according to RaxML (Msp_0101 being the exception; see Table 1). Similarly, *M. stadtmanae’*s closest phylogenetic neighbor, *M. smithii*, has 29 annotated glycosyltransferases, 22 of which have been previously indicated as being of bacterial origin (Lurie-Weinberger et al., 2011). Interestingly, when comparing the glycosyltransferase content of these two methanogens, only eight are shared by both. Of these eight, half were indicated to be LGT candidates in *M. smithii* (Lurie-Weinberger et al., 2011). It has been noted that “Both *M. smithii* and *M. stadtmanae* dedicate a significantly larger proportion of their “glycobiome” to GT2 family glycosyltransferases than any of the sequenced non-gut associated methanogens”(Samuel et al., 2007), and these glycosyltransferases therefore are believed to play an important role in the adaptation of these methanogens to their environment. It is also interesting to note in this regard that these glycosyltransferases in both *M. smithii* and *M. stadtmanae* are greatly diverse, with great differences both in terms of size (ranging from 190 to 1193 amino acids in length) and sequence (in *M. smithii*, for example, the 17 glycosyltransferases included in the LGT-rich genomic region shared less than 10% identity to one another). Therefore it is unlikely that this abundance of glycosyltransferases is the result of a transfer followed by duplication of these proteins, but rather the transfer or co-transfer of multiple, different, glycosyltransferases.

**Table 1:** **Putative glycosyltransferases present in *M. stadtmanae*, and their inferred origin**.

Gene	Putative origin	Direction	Raxml	Direction	*M. smithii* homolog	LGT
Msp_0039	LGT	Bacteria → Archaea	LGT	Bacteria → Archaea	None	
Msp_0042	LGT	Bacteria → Archaea	LGT	DU	None	
Msp_0044	LGT	DU	LGT	DU	None	
Msp_0045	Non-LGT	N/A	N/A	N/A	None	
Msp_0049	Non-LGT*	N/A	N/A	N/A	None	
Msp_0051	Non-LGT	N/A	N/A	N/A	None	
Msp_0052	Non-LGT	N/A	N/A	N/A	None	
Msp_0053	Non-LGT	N/A	N/A	N/A	None	
Msp_0054	Non-LGT	N/A	N/A	N/A	None	
Msp_0055	LGT	Bacteria → Archaea	LGT	Bacteria → Archaea	None	
Msp_0056	Non-LGT	N/A	N/A	N/A	None	
Msp_0057	Non-LGT	N/A	N/A	N/A	None	
Msp_0080	Non-LGT	N/A	N/A	N/A	None	
Msp_0101	LGT	Archaea → Bacteria	non-LGT	N/A	Msm_0836	Non-LGT
Msp_0203	LGT	DU	LGT	DU	None	
Msp_0206	LGT	DU	LGT	DU	Msm_1329	LGT
Msp_0207	LGT	Bacteria → Archaea	LGT	Bacteria → Archaea	Msm_1330	LGT
Msp_0212	Non-LGT	N/A	N/A	N/A	None	
Msp_0215	LGT	DU	LGT	DU	None	
Msp_0218	LGT	Bacteria → Archaea	LGT	Bacteria → Archaea	None	
Msp_0220	Non-LGT	N/A	N/A	N/A	None	
Msp_0441	LGT	Bacteria → Archaea	LGT	Bacteria → Archaea	None	
Msp_0442	LGT	Bacteria → Archaea	LGT	Bacteria → Archaea	Msm_1594	LGT
Msp_0492	LGT	DU	LGT	N/A	Msm_1313	Non-LGT
Msp_0493	LGT	Bacteria → Archaea	LGT	Bacteria → Archaea	None	
Msp_0495	LGT	DU	LGT	DU	None	
Msp_0496	LGT	DU*	LGT	DU*	None	
Msp_0500	LGT	DU*	LGT	DU*	Msm_1312	LGT
Msp_0538	LGT	DU	LGT	DU	None	
Msp_0541	Non-LGT	N/A	N/A	N/A	Msm_1623	Non-LGT
Msp_0645	Non-LGT	N/A	N/A	N/A	Msm_0423	Non-LGT
Msp_0989	Non-LGT	N/A	N/A	N/A	None	
Msp_0991	Non-LGT	N/A	N/A	N/A	None	
Msp_1087	Non-LGT	N/A	N/A	N/A	None	

*DU, direction unknown*.

DU*, direction unknown, but *M. stadtmanae* and *M. smithii* are the only Archaea on the tree.

**Figure 3:**
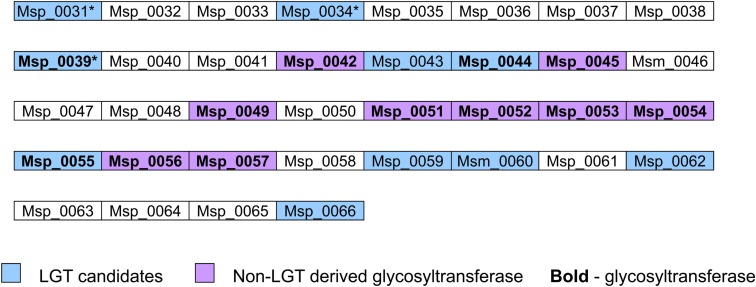
**The Msp_0031-Msp_0066 genomic region, encoding glycosyltransferase and LGT candidates**.

**Figure 4:**
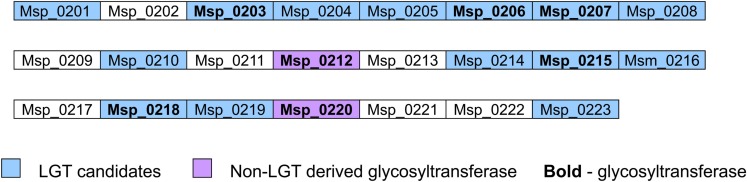
**The Msp_0201-Msp_0223 genomic region encoding glycosyltransferase and LGT candidates**.

Interestingly, most glycosyltransferases-encoding genes are not evenly distributed throughout the genome of *M. stadtmanae*, but rather are found in clusters. The largest group of glycosyltransferases, containing 12 separate genes, can be found in the genomic region between Msp_0031-Msp_0066, spanning over 36 genes. This region is also rich in laterally transferred genes, as 10 of these proteins have been identified as LGT candidates, including three of the glycosyltransferases (see Figure 3). Another such region is located between Msp_0201-Msp0223, which includes seven glycosyltransferases, five of which are also LGT candidates. This locus includes in total 14 LGT candidates (see Figure 4). Msp_0201 itself is a putative recombinase. This situation has also been observed in *M. smithi*, which has two large genomic regions rich in both glycosyltransferases and LGT candidates (Msm_1278-Msm_1331 with 33 LGT candidates and 17 glycosyltransferases, and Msm_1502-Msm_1545 with 24 LGT candidates and three glycosyltransferases). Thus, mammalian-associated methanogens tend to have glycosyltransferases that are horizontally derived.

### Multiple ABC transporters that have been derived from LGT in *M. stadtmanae*

The active transport capability of *M. stadtmanae* has been greatly influenced by bacterial-derived genes. Out of 22 annotated ABC transporter components in *M. stadtmanae*, 11 have been acquired by LGT (see Table 2). LGT candidates of bacterial origin include all three polar amino acid ABC transporter components (Msp_0958-Msp_0960, an ATP-binding protein, a permease, and a periplasmic substrate-binding protein, respectively), two of three annotated nitrate/sulfonate/bicarbonate transport system ABC transporter components (Msp_1000-Msp_1001, an ATP-binding protein, and a permease), and both annotated polysaccharide/polyol phosphate ABC transporter components (Msp_0204-Msp_0205, ATP-binding protein, and permease). LGT candidates with no clearly inferred direction of transfer include three out of 10 annotated peptide ABC transporter proteins (Msp_0808-Msp_0809 and Msp_0847, all ATP-binding proteins) and another LGT candidate of this class is the molybdenum ABC transporter ATP-binding protein (see Table 2).

**Table 2:** **Transport-related proteins acquired by LGT**.

Gene	Annotation	Fraction	Direction (distance matrix)	Direction (RaxML)	*M. smithii* homolog	Annotation	
Msp_0204	Polysaccharide/polyol phosphate ABC transporter permease	2/2	Bacteria → Archaea	Bacteria → Archaea	Msm_1325	Polysaccharide/polyol phosphate ABC transporter, permease component	LGT
Msp_0205	Polysaccharide/polyol phosphate ABC transporter ATP-binding protein		Bacteria → Archaea	Bacteria → Archaea	Msm_1326	Polysaccharide/polyol phosphate ABC transporter, ATPase component	LGT
Msp_0610	Molybdenum ABC transporter ATP-binding protein	1/1	Direction unknown*	Direction unknown*	Msm_0593	Multidrug ABC transporter, ATPase component, CcmA	LGT
Msp_0808	Peptide ABC transporter ATP-binding protein	2/5	Direction unknown	No LGT	Msm_0304	Peptide/nickel ABC transporter, ATP-binding component, DppF	LGT
Msp_0809	Peptide ABC transporter ATP-binding protein		Direction unknown	No LGT	Msm_0303	Peptide/nickel ABC transporter, ATP-binding component, DppD	NO
Msp_0810	ABC-type dipeptide transport system, permease protein		No LGT*	No LGT*	Msm_0302	Peptide/nickel ABC transporter, permease component, DppC	NO
Msp_0811	ABC-type dipeptide transport system, permease protein		No LGT*	No LGT*	Msm_0301	Peptide/nickel ABC transporter, permease component, DppB	NO
Msp_0812	ABC-type dipeptide transport system, dipeptide-binding protein		No LGT*	No LGT*	Msm_0300	Peptide/nickel ABC transporter, solute-binding component	NO
Msp_0847	Peptide ABC transporter ATP-binding protein	1/5	Direction unknown	Direction unknown	N/A	N/A	N/A
Msp_0848	Peptide ABC transporter ATP-binding protein		No LGT*	No LGT*	N/A	N/A	N/A
Msp_0849	Peptide ABC transporter permease		No LGT*	No LGT*	N/A	N/A	N/A
Msp_0850	Peptide ABC transporter permease		No LGT*	No LGT*	N/A	N/A	N/A
Msp_0851	Peptide ABC transporter solute-binding protein		No LGT*	No LGT*	N/A	N/A	N/A
Msp_0958	Polar amino acid ABC transporter ATP-binding protein	3/3	Bacteria → Archaea	Bacteria → Archaea	Msm_0805	Polar amino acid ABC transporter, ATPase component	LGT
Msp_0959	Polar amino acid ABC transporter permease		Bacteria → Archaea	Bacteria → Archaea	Msm_0806	Polar amino acid ABC transporter, permease component	LGT
Msp_0960	Polar amino acid ABC transporter periplasmic substrate-binding protein		Bacteria → Archaea	Bacteria → Archaea	Msm_0807	Polar amino acid ABC transporter, substrate-binding component	LGT
Msp_1000	Nitrate/sulfonate/bicarbonate transport system ABC transporter ATP-binding protein	2/3	Bacteria → Archaea	Direction unknown	Msm_0290	Nitrate/sulfonate/bicarbonate ABC transporter, ATPase component, TauB	LGT
Msp_1001	Nitrate/sulfonate/bicarbonate ABC transporter permease		Bacteria → Archaea	Direction unknown	Msm_0291	Nitrate/sulfonate/bicarbonate ABC transporter, permease component,TauC	NO

No LGT* represent a case where *Methanosarcina* is the closest phylogenetic neighbor in an otherwise bacterial clade.

It is interesting to note that while our analysis has indicated Msp_0808-Msp_0809 to be LGT-derived components of a peptide ABC transporter, the other putative components of this transporter were not detected as such. Similarly, though only Msp_0847 was detected as an LGT candidate which is a component of a peptide ABC transporter, the adjacent genes Msp_0848-Msp_0851 that should encode the remaining peptide ABC transporter components did not have bacterial sister taxa. This may indicate that genes encoding transporter components were acquired and replaced ancestral components, or, more likely that our definition of LGT can occasionally be too conservative. Indeed, the first two components Msp0808-Msp0809 have been indicated to be involved in LGT, while three other components of the same ABC transporter, Msp_0810-0812 are not. Yet, on closer inspection of the three non-LGT trees, it becomes clear that the only reason that they were not indicated as transfer events is that their closest phylogenetic neighbor is the archaeon *Methanosarcina*, which appears as closest phylogenetic neighbor in all three trees. *Methanosarcina* belongs to a different methanogenic order, very distant from the Methanobacteriales order, to which *Methanobrevibacter* and *Methanosphaera* belong, and so a gene shared exclusively by these taxa among all archaea has probably been gained twice by these distant lineages. However, *Methanosarcina*, *Methanobrevibacter*, and *Methanosphaera* are all nested in a clearly bacterial clade, and the most parsimonious scenario is that all five components were therefore acquired through inter-domain LGT from bacteria into archaea. Moreover, the reason that we could not infer the direction of transfer of the two components that were identified as involved in LGT was also the result on the existence of a *Methanosarcina* homolog on an otherwise clearly bacterial tree. Taken together these findings imply that all five components of this ABC transporter are of bacterial origin. This example shows that while it is generally advisable to use a relatively stringent approach of phylogenetic LGT detection, the number of LGT event detected is probably but a lower bound on the actual number of such events that have occurred. This is especially true for cases involving *Methanosarcina*, which has acquired many bacterial genes laterally (Deppenmeier et al., 2002). The same is true for the second ABC transporter mentioned, Msp_0847-Msp_0851, since its first component Msp_0847 has been indicated as a LGT candidate for which no direction could be inferred, when in fact it is the presence of *Methanosarcina* in an otherwise bacterial clade that is the sole reason for this status, while the other four components Msp_0848-Msp_0851 are all considered non-LGTs only because of the presence of *Methanosarcina* as a sister taxon on the tree. Therefore, this ABC transporter as well is probably the result of a transfer event from a bacterial source.

*Methanobrevibacter smithii*, *M. stadtmanae*’s closest phylogenetic neighbor, has 49 annotated ABC transporter components, 26 of which have been acquired from bacteria. These also include components of the Polysaccharide/polyol phosphate ABC transporter, of the polar amino acid ABC transporter, nitrate/sulfonate/bicarbonate ABC transporter, and of peptide ABC transporter components (Lurie-Weinberger et al., 2011), yet other ABC transporters that have been abundant in *M. smithii* such as components of the cobalt ABC transporter are completely missing from the genome of *M. stadtmanae*. Cobalt is a critical micronutrient for growth of many microorganisms, including methanogens, where it is required for the synthesis of vitamin B12 and methylcobalamine, a co-enzyme involved in methanogenesis. *M. stadtmanae*, unlike *M. smithii*, has a highly limited energy metabolism (Fricke et al., 2006). This human intestinal inhabitant can generate methane only by reduction of methanol with H_2_ and is dependent on acetate as a carbon source (Fricke et al., 2006). Thus, the difference in cobalt-uptake proteins may result in an inferior methanogenesis capability in *M. stadtmanae* compared to *M. smithii*, which may be better adapted to the colonic milieu. In addition to cobalt-uptake, *M. stadtmanae* is also missing the laterally acquired adhesin-like proteins, which contribute greatly to surface functions in *M. smithii*. One may speculate that the lack of these genetic features is one of the reasons that *M. stadtmanae* is much less abundant in the human gut microbiome than *M. smithii*, which seems to be better adapted to this niche (Dridi et al., 2009; Lurie-Weinberger et al., 2011).

### Gene content reconstruction of an ancestral mammal-symbiotic methanogen

**Figure 5:**
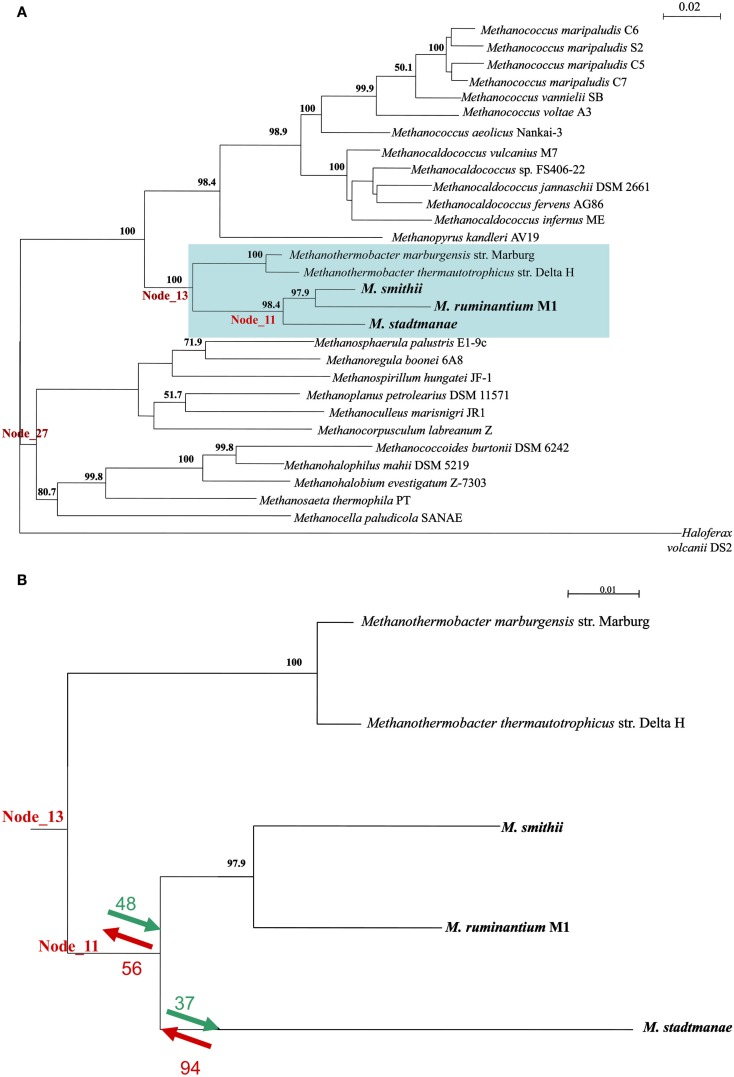
**(A)** A 16S ribosomal gene tree representing the phylogenetic relationship between the Archaea in the ACE analysis. Numbers represent% of bootstrap support for nodes exceeding 50%. **(B)** The subtree representing nodes 11 and 13 and the COGs that were either gained (green) or lost (red) upon their divergence, and the divergence of *M. stadtmanae* and the two *Methanobrevibacter* species. A complete list of species can be found in Table S4 in Supplementary Material.

To better understand the contribution of LGT to the evolutionary processes leading to the emergence of the extant mammal-symbiotic methanogenic Archaea (*M*. *stadtmanae*, *M. smithii*, and *M. ruminantium*) a COG-based analysis was performed. This analysis identifies which COGs were acquired and which were lost in these three genomes as opposed to the prior “ancestral” class I methanogen (i.e., the ancestor of the orders Methanopyrales, Methanobacteriales, and Methanococalles, see Luo et al., 2009) they have evolved from. This was done using the ACE approach (Tuller et al., 2010), which uses co-evolution data to infer gene content of ancestral taxa. For that end, a phylogenetic tree was first constructed using the 16S ribosomal gene of 29 methanogenic Archaea and the 16S of the non-methanogenic halophilic Archaea *Haloferax volcanii*_DS2. This tree and the COG content of extent methanogens were used to deduce COG content of genomes in node_11 (representing the common ancestor of the three mammal-symbiotic methanogens) to its progenitor node_13, which represents the ancestral COG content of node 11 and two more distantly related *Methanothermobacter* genomes, *Methanothermobacter marburgensis* str. Marburg and *Methanothermobacter thermautotrophicus* str. Delta (see Figure 5; Table S4 in Supplementary Material).

Both gene gain and gene loss played important roles in the evolution of the gene content of the ancestral mammal-symbiotic methanogen: 48 COGs were acquired, while 56 were lost. The most abundant gene functions gained (10/48) were ABC transporters, prominently the nickel/peptide ABC transporter (which includes DppB-D and DppF in *M. smithii* and its homologs in *M. stadtmanae*). Six of 10 gained ABC transporters in *M. smithii* and six of 10 in *M. stadtmanae* were indicated as LGT candidates by our analyses (with COG1134 representing two separate genes in *M. smithii*, Msm_1592 and Msm_1326) and therefore further support our finding that these six genes are derived from LGT from outside the methanogenic archaea. The remaining four genes might represent other LGT events that are so ancient that they have lost the phylogenetic signal that would enable their identification as LGT-products.

There are 56 COGs that are present in node 13, and are absent in node 11, meaning that they are ancestral COGs that have been lost in one or more of the three mammal-associated methanogens. These 56 COGs include three ABC transporters (two missing from both *M. smithii* and *M. stadtmanae*, while one COG1668 is present in *M. smithii*), five predicted membrane proteins, and several metabolism-related proteins (Table S5 in Supplementary Material; Figure 5B). As mentioned above, not all 56 proteins are completely missing from all three methanogens, and five of these 56 genes are present in *M. smithii* but are absent from both the *M. stadtmanae* and *M. ruminantium* genomes. These five COGs are thus likely to have been acquired by a lineage leading to *M. smithii* after the split from *M. stadtmanae*, and include a multidrug ABC transporter, permease component, a metal-dependent hydrolase, ornithine cyclodeaminase and two hypothetical proteins. The ABC transporter protein, Msm_1484, has already been indicated in previous analyses to have been laterally transferred from Bacteria into Archaea (Lurie-Weinberger et al., 2011) and is as part of a large nine-component transporter with seven of its nine proteins separately indicated to have been the result of LGT (Table S5 in Supplementary Material).

If we focus on changes that have occurred after the divergence of *M. stadtmanae* from the other two mammalian-associated methanogens, i.e., if we compare node_11 to the COG content of *M. stadtmanae*, it is clear that here too both gain and loss have played a part in the evolution of this genome. Thirty-seven COGs have been gained and 94 lost in *M. stadtmanae* compared to the two *Methanobrevibacter* species. COGs gained include mostly hypothetical proteins (17 proteins), but also two restriction-modification system proteins known to be frequently mobile (Ishikawa et al., 2010; Table S6 in Supplementary Material). COGs lost include eight ABC transporter components; three Fe^3+^-related transport components (cobalamin/Fe^3+^ -siderophores periplasmic component, ATPase component, Fe^3+^-hydroxamate, and Fe^3+^ -siderophore permease component) two molybdate-transport components (ATPase and periplasmic components), an Na+ efflux pump, permease component, an antimicrobial peptide ATPase component, and ABC transporter involved in lipoprotein release, permease component (Table S6 in Supplementary Material; Figure 5B).

From a broader perspective, when comparing the common ancestral methanogen as represented in node 27 to ancestral mammal-associated methanogen, we can observe that 157 COGs have been acquired while 194 genes were lost in the latter lineage. These include gain of 16 membrane associated proteins, five separate ABC transporter components that comprise parts of four different transporters (two amino acid transport, a polar amino acid transport, a dipeptide/oligopeptide/nickel and the molybdate transporter component,) and two pilus assembly proteins TadB and CpaF (Table S7 in Supplementary Material).

COGs lost, on the other hand, include many CO dehydrogenase/acetyl-CoA synthase-related proteins, including CO dehydrogenase/acetyl-CoA synthase alpha to epsilon subunits (COG1152, COG1614, COG1456, COG2069, and COG1880 respectively) and CO dehydrogenase maturation factor (COG3640). Also, nine membrane associated proteins and six ABC transporter component proteins, which include two tungstate transport components, a metal ion transporter, a Mn^2+^/Zn^2^ transport systems ATPase component, a Mn^2+^/Zn^2+^ transport permease component, and a sulfate transporter component (Table S7 in Supplementary Material). Thus, the ancestral mammalian-associated methanogen had lost some classic autotrophic capabilities and gained better ways to import organic substrates from its, now-richer, environment.

Both codon analyses and the ancestral gene content reconstruction support the same scenario, in which ancient LGT, from anaerobic bacteria and possibly other archaea facilitated niche adaptation relatively early in the evolution of the mammal-associated methanogenic genera *Methanobrevibacter* and *Methanosphaera*. In both of these genera the largest fraction of the laterally acquired genes originated from the Firmicutes, with fewer than expected genes coming from the other common dominant intestinal phylum, the Bacteroidetes. This could imply that there unknown factors, such as mobile elements that somehow facilitate genetic exchange between Firmicutes and members of the Methanobacteriales. However, a more likely explanation is that most gene transfer events had taken place in a pre-mammalian niche where Firmicutes were even more dominant, and this could explain why many laterally acquired genes are still shared between intestinal and ruminal *M. smithii and M. ruminantium* genomes (Lurie-Weinberger et al., 2011), despite the ecological differnces between these two host niches.

## Conflict of Interest Statement

The authors declare that the research was conducted in the absence of any commercial or financial relationships that could be construed as a potential conflict of interest.

## Supplementary Material

The Supplementary Material for this article can be found online at: http://www.frontiersin.org/Evolutionary_and_Population_Genetics/10.3389/fgene.2012.00182/abstract

Supplementary Table S1 **All LGT candidates in *M. stadtmanae***.

Supplementary Table S2 **ENC and CAI values for all LGT candidates in *M. stadtmanae***.

Supplementary Table S3 **ENC and CAI averages of all LGT candidates, and of the entire genome of *M. stadtmanae***.

Supplementary Table S4 **All archaeal species used in the reconstruction of COG content of the “ancestral” methanogen**.

Supplementary Table S5 **All COGs gained and lost in the three mammalian-associated methanogens (Node_11) compared to the its progenitor node (node_13)**.

Supplementary Table S6 **All COGs gained and lost in *M. stadtmanae* compared to the ancestor of the three mammalian associated methanogens (Node_11)**.

Supplementary Table S7 **All COGs gained and lost in the three mammalian associated methanogens (Node_11) compared to the “ancestral” methanogen (Node_27)**.
